# A systematic review of robot-assisted anti-reflux surgery to examine reporting standards

**DOI:** 10.1007/s11701-022-01453-2

**Published:** 2022-09-08

**Authors:** Marc M. Huttman, Harry F. Robertson, Alexander N. Smith, Sarah E. Biggs, Ffion Dewi, Lauren K. Dixon, Emily N. Kirkham, Conor S. Jones, Jozel Ramirez, Darren L. Scroggie, Benjamin E. Zucker, Samir Pathak, Natalie S. Blencowe, RoboSurg collaborative group, Barry G. Main, Jane M. Blazeby, Ben Zucker, Sarah Dawson, Abi Vallance, Aimee Wilkinson, Amber Young, Annabel Jones, Aya Abbas, Benedict Turner, Charlie Thomas, Christin Hoffmann, David Henshall, Eleanor Boden, Emma Gull, Emma Sewart, Fergus Wood, Francesca Loro, Freya Hollowood, George E. Fowler, George Higginbotham, Grace Sellers, Ioan Hughes, Ishita Handa, Jonathan Rees, Lorna Leandro, Louisa Paynter, Lucy Huppler, Lysander Gourbault, Manuk Wijeyaratne, Max Dewhurst, Max Shah, Miraen Kiandee, Mo Dada, Oliver Brewster, Pat Lok, Rahul Winayak, Reesha Ranat, Rhiannon Macefield, Ruby Lawrence, Ryan Millar, Sam Lawday, Sanjush Dalmia, Sian Cousins, Sophie Rozwadowski, Tanya Robinson, Teresa Perra, Tjun Wei Leow, Tom Brankin-Frisby, Will Baker, William Hurst, Ysabelle Embury Young, Christin Hoffman, Hollie S. Richards, James Olivier, Keng Siang Lee, Rory Purves

**Affiliations:** 1 Centre for Surgical Research, Population Health Sciences, Bristol Medical School, University of Bristol, Canynge Hall, 39 Whatley Road, Bristol, BS8 2PS UK; 2 University College Hospital, University College London Hospitals NHS Foundation Trust, London, UK; 3 St. Mary’s Hospital, Imperial College Healthcare NHS Trust, London, UK; 4 University of Exeter Medical School, Exeter, UK; 5 University Hospitals Bristol and Weston NHS Foundation Trust, Bristol, UK; 6 Musgrove Park Hospital, Somerset NHS Foundation Trust, Taunton, UK; 7 Torbay Hospital, Torbay and South Devon NHS Foundation Trust, Torquay, UK; 8 St James’s University Hospital, Leeds Teaching Hospitals NHS Trust, Leeds, UK

**Keywords:** Robotic surgery, Anti-reflux surgery, Fundoplication, Outcome reporting, IDEAL framework

## Abstract

Robot-assisted anti-reflux surgery (RA-ARS) is increasingly being used to treat refractory gastro-oesophageal reflux disease. The IDEAL (Idea, Development, Exploration, Assessment, Long-term follow up) Collaboration’s framework aims to improve the evaluation of surgical innovation, but the extent to which the evolution of RA-ARS has followed this model is unclear. This study aims to evaluate the standard to which RA-ARS has been reported during its evolution, in relation to the IDEAL framework. A systematic review from inception to June 2020 was undertaken to identify all primary English language studies pertaining to RA-ARS. Studies of paraoesophageal or giant hernias were excluded. Data extraction was informed by IDEAL guidelines and summarised by narrative synthesis. Twenty-three studies were included: two case reports, five case series, ten cohort studies and six randomised controlled trials. The majority were single-centre studies comparing RA-ARS and laparoscopic Nissen fundoplication. Eleven (48%) studies reported patient selection criteria, with high variability between studies. Few studies reported conflicts of interest (30%), funding arrangements (26%), or surgeons’ prior robotic experience (13%). Outcome reporting was heterogeneous; 157 distinct outcomes were identified. No single outcome was reported in all studies.The under-reporting of important aspects of study design and high degree of outcome heterogeneity impedes the ability to draw meaningful conclusions from the body of evidence. There is a need for further well-designed prospective studies and randomised trials, alongside agreement about outcome selection, measurement and reporting for future RA-ARS studies.

**Supplementary Information:**

The online version contains supplementary material available at 10.1007/s11701-022-01453-2.

## Introduction

Laparoscopic anti-reflux surgery is considered the standard treatment for refractory gastro-oesophageal reflux disease (GORD), conferring a shorter length of hospital stay, fewer complications and quicker return to baseline function than the equivalent open technique [1–3]. Robot-assisted anti-reflux surgery (RA-ARS) is a recent surgical innovation which may offer improved ergonomics, dexterity, and three-dimensional vision, all of which are particularly useful around the oesophageal hiatus [4–6]. Whether these theoretical advantages translate into better patient outcomes remains unclear. The Cumberlege report (also known as the ‘First do no harm’ report) recommended that innovative procedures (such as RA-ARS) should be robustly evaluated before being widely adopted, with transparent communication with patients about the risks, benefits and alternatives [7]. Whilst RA-ARS has been adopted by certain NHS service providers, it remains unclear whether it has been robustly evaluated.

Evaluating surgical innovations is challenging due to idiosyncrasies related to surgical practice [8]. New procedures are modified case-to-case, and may continue to be modified even after becoming widely adopted, leading to uncertainty about the optimal timing of randomised controlled trials (RCTs). Operator learning curves and a lack of standard outcome measures create additional complexity [9]. To address these challenges, the IDEAL (Idea, Development, Exploration, Assessment, and Long-term follow-up) Collaboration proposed a staged approach for evaluating and reporting surgical innovations [10]. The IDEAL framework progresses from Stage 1 (first-in-human) to Stage 4 (Long-term follow-up of established techniques), and makes recommendations about methodology, governance factors, ethical factors, and the quality of reporting for each stage. The level of understanding of these 2009 recommendations was found to be low [11], leading to subsequent publication of practical guidance [9], updates [12] and reporting guideline checklists [13] to further improve the quality of reporting in surgical innovation.

High quality reporting of surgical innovation has important benefits. It supports shared learning between innovators, facilitating diffusion of successful techniques as well as timely elimination of poor techniques, thereby improving research efficiency and avoiding the repeated exposure of patients to harmful interventions [14]. High quality reporting also permits better comparison between studies [9], leading to meaningful meta-analyses that may inform clinical practice guidelines. RA-ARS was first described in 2001 [15], with subsequent increases in both volume of publications and technique adoption. Despite the quantity of research, the *quality* of reporting in these studies has never to our knowledge been evaluated. The aim of this study was therefore to evaluate the standard to which RA-ARS has been reported during its evolution, in relation to the IDEAL framework. The study did not aim to examine effectiveness or efficacy of RA-ARS.

## Methods

The systematic review was informed by previously published studies [16, 17], and is summarised below.

### Search strategy

Systematic searches using terms for ‘anti-reflux’ and ‘robotic surgery’ were performed in OVID (MEDLINE, Embase), Cochrane Library and Web of Science (SCI-EXPANDED, ESCI) from inception to June 2020 (Supplementary Table 1).

### Study eligibility

All primary research studies pertaining to RA-ARS in adults with symptomatic GORD were included. Studies relating to paraoesophageal or giant hernias were excluded because of differences in operative techniques and disease and complication profiles. Studies where the indication for surgical intervention was not primarily for symptoms of GORD (e.g. Roux-en-Y gastric bypass with concomitant hiatus hernia repair) were excluded. Studies reporting combined interventions in which the outcomes could not be separated were excluded (e.g. those describing a centre’s experience of robotic surgery across multiple specialties and procedures). Conference abstracts and non-English language studies were excluded [18].

### Study selection

After de-duplication, abstracts were screened by two independent reviewers (MH and HR). Full texts were retrieved and screened for eligibility in the same manner. Conflicts at both stages were resolved by discussion involving a third independent reviewer (NB), providing a final list of included papers.

### Data extraction

Data extraction was undertaken by two independent reviewers using a purpose-built online tool. After appropriate training, primary data extraction was performed by a member of the ‘RoboSurg Collaborative’ (see participating investigators). Data were then verified by a senior member of the research team. Disagreements were discussed with a third independent reviewer where necessary (NB). Data categories were informed by IDEAL guidelines and a previously published study [17].

#### General study characteristics and IDEAL stage

Data were collected on the publication year, country of origin, study design, whether prospective or retrospective, number of participants, and number and type of included centres (e.g. specialist, general). Information about the intervention (and, where applicable, comparator group(s)) was noted. Where reported, the IDEAL stage of the study was recorded. Studies that did not provide this information were classified into IDEAL stages by two researchers (MH and HR), using an algorithm created by the IDEAL collaboration [19]. The first case report [4] was considered to be the stage 1 first-in-human study. Bias in the included RCTs was assessed using the Cochrane risk of bias (ROB2) tool [20].

#### Patient selection and demographics

All reported inclusion and exclusion criteria were extracted, as well as statements about what happened to patients not meeting the inclusion criteria. All reported demographics of the included patients (e.g. age, sex and co-morbidities) were also recorded.

#### Governance and ethical factors

Statements confirming institutional review board (IRB) or ethics committee approval were documented. Reports of patient consent, including those specifically regarding to the innovative nature of RA-ARS, were recorded verbatim. Funding and conflicts of interest (COI) declarations were noted.

#### Surgeon expertise and training

Any prespecified requirements for study participation, such as experience or training courses, were recorded. The number of surgeons participating in each study was documented along with their respective grades (e.g. trainee/junior or consultant/attending). Information regarding the reporting or measurement of surgeons’ learning curves was extracted verbatim.

#### Outcome selection, measurement and reporting

Individual outcomes from each study were extracted verbatim and coded by two independent reviewers (MH and HR) into one of seven pre-determined domains (technical outcomes, complications, investigations, persistence of symptoms, patient-reported outcomes, surgeon-reported outcomes, and health economic outcomes—Supplementary Table 2). The total number of distinct outcomes across all studies and the total number of outcomes reported in each domain were recorded. Outcomes with the same meaning that were worded differently, for example ‘length of stay’ and ‘duration of hospitalisation’, were not counted as distinct. Where available, the follow-up period for each recorded outcome was documented. If a core outcome set (i.e. an agreed minimum set of outcomes that should be reported in all clinical trials of a specific disease [21]) was used, this was noted.

### Data synthesis

Data were summarised in a narrative synthesis and descriptive statistics were used where appropriate. Meta-analyses were not performed as we aimed to examine the reporting of RA-ARS, rather than its efficacy or effectiveness. Sequential progression of data categories through the IDEAL stages was displayed graphically where appropriate.

## Results

### Included studies

**Fig. 1:**
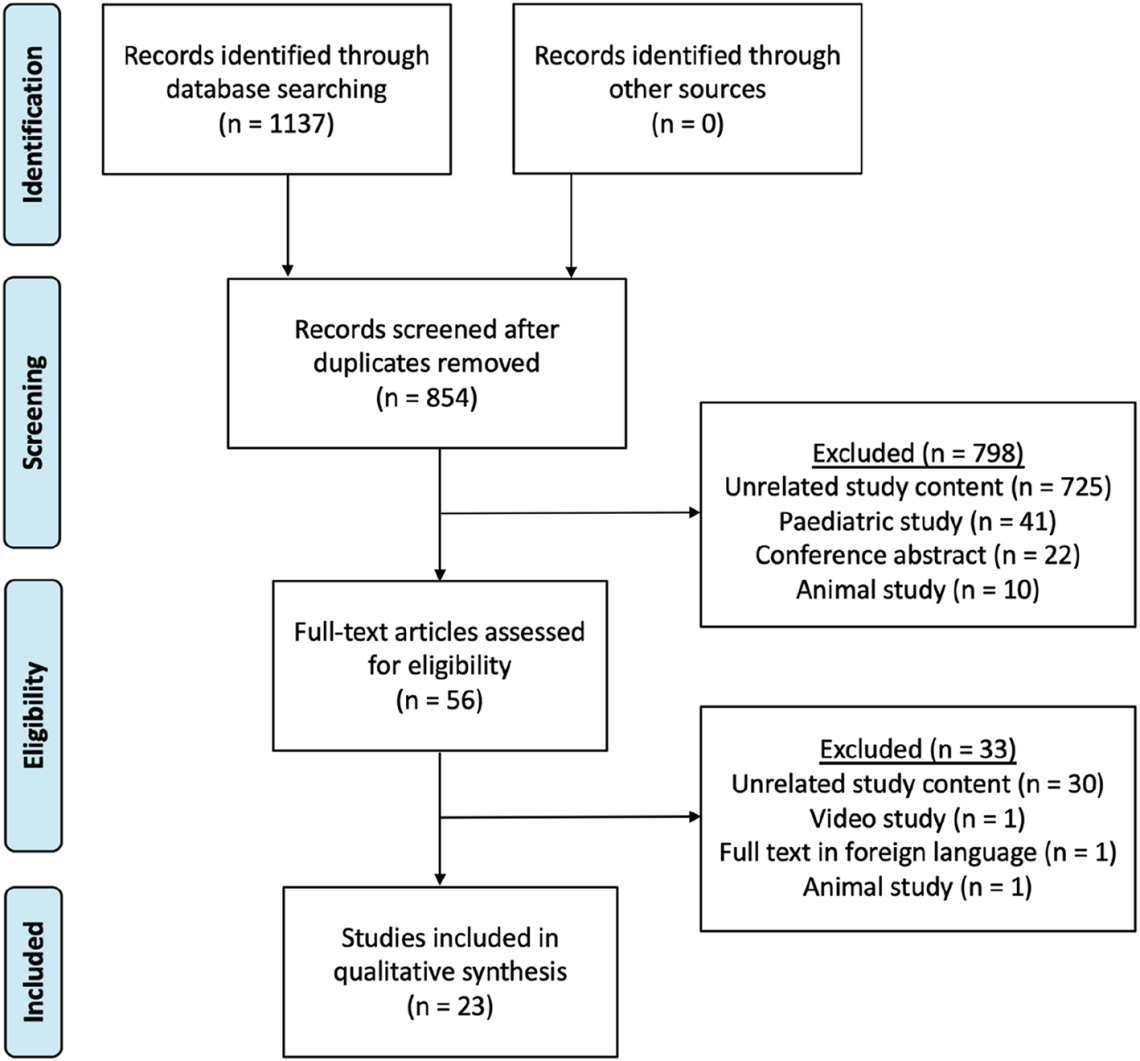
PRIMSA diagram showing selection of articles for review

A total of 854 abstracts were screened and 56 full texts were assessed for eligibility. Of these, 23 studies were included in the analysis [4–6, 15, 22–40] (Fig. 1).

### General study characteristics and IDEAL stage

A total of 13,506 participants were included across the 23 studies (range, 1–12,079; median, 44; Table 1). Of these, 741 participants underwent RA-ARS, 10,597 underwent L-ARS and 2168 underwent an open procedure. Most studies (*n* = 17, 74%) included fewer than 100 participants. Studies were published between 2001 and 2019 and included two case reports (9%), five case series (22%), 10 comparative cohort studies (43%) and six randomised controlled trials (RCTs, 26%). Of the 18 (78%) studies reporting the temporality of data collection, 10 (43%) were prospective and 8 (35%) were retrospective. Most were single-centre studies (*n* = 11, 48%) and were undertaken in the USA (*n* = 7, 30%), although 9 (39%) did not provide this information. The type of participating centre was generally omitted (*n* = 18, 78%).

**Table 1:** Characteristics of included studies

Paper number	Author	Year	Country	Study type	Data collection	IDEAL Stage^a^	Number of participants	Procedure of interest	Comparison arms (participants per arm)	Number and type of centre
1	Chapman et al. [4]	2001	United States	CR	NR	1	1	Nissen	N/A	Single centre. Type NR
2	Cadière et al. [15]	2001	Mexico	RCT	Prospective	3	21	Nissen	RA-ARS (10) vs L-ARS (11)	Single centre. Type NR
3	Melvin et al. [6]	2002	United States	Co	Prospective	2b	40	Nissen & Toupet	RA-ARS (20) vs L-ARS (20)	NR
4	Wykypiel et al. [22]	2003	Austria	CS	NR	2a	9	Toupet	N/A	NR
5	Braumann et al. [23]	2005	Germany	CS	NR	2a	6	Dor	N/A	NR
6	Draaisma et al. [5]	2006	Netherlands	RCT	Prospective	3	50	Nissen	RA-ARS (25) vs L-ARS (25)	Single centre. Type NR
7	Morino et al. [25]	2006	Italy	RCT	Prospective	3	50	Nissen	RA-ARS (25) vs L-ARS (25)	NR
8	Nakadi et al. [24]	2006	Belgium	RCT	Prospective	3	20	Nissen	RA-ARS (9) vs L-ARS (11)	Specialist centre. Number NR
9	Heemskerk et al. [26]	2007	Denmark	Co	Retrospective	2b	22	Nissen	RA-ARS (11) vs L-ARS (11)	Single centre. Type NR
10	Muller-Stich et al. [27]	2007	Germany, Romania	RCT	Prospective	3	40	Nissen	RA-ARS (20) vs L-ARS (20)	NR
11	Dunnican et al. [28]	2008	United States	Co	Retrospective	2b	26	Nissen	RA-Nissen (7) vs RA-paraoesophageal hernia repair (19)	Single centre. Type NR
12	Hartmann et al. [29]	2008	Germany	CS	Prospective	2b	118	Dor & Toupet	N/A	NR
13	Ceccarelli et al. [30]	2009	Italy	Co	Retrospective	2b	182	Nissen	RA-ARS (45) vs L-ARS (137)	Single centre. Type NR
14	Hartmann et al. [31]	2009	Germany	Co	Prospective	2b	80	Dor	RA-ARS (18) vs L-ARS (62)	Single, specialist centre
15	Muller-Stich et al. [32]	2009	Germany	RCT	Prospective	3	40	Nissen	RA-ARS (20) vs L-ARS (20)	Single centre. Type NR
16	Franzzoni et al. [33]	2012	Italy	Co	Retrospective	2b	88	Nissen	RA-ARS (44) vs L-ARS (44)	Single centre. Type NR
17	Franzzoni et al. [34]	2013	Italy	CS	NR	2b	44	Not recorded	N/A	Single centre. Type NR
18	Owen et al. [35]	2014	United States	Co	Retrospective	2b	12,079	Nissen	RA-ARS (339) vs L-ARS (9572) vs Open ARS (2168)	Multi centre. Specialist centres
19	Jensen et al. [37]	2016	Denmark	Co	Retrospective	2b	103	Nissen & Toupet	RA-ARS (39) vs L-ARS (64)	Multi centre. Type NR
20	Andrade et al. [36]	2017	United States	CR	NR	1	1	Dor	N/A	NR
21	Moore et al. [38]	2017	United States	Co	Retrospective	2b	105	Nissen	RA-ARS (58) vs L-ARS (47)	Single, specialist centre
22	Gharagozloo et al. [39]	2019	USA, Netherlands	CS	Retrospective	2b	313	Belsey	N/A	Multi centre, both specialist and general
23	Giovannetti et al. [40]	2019	United States	Co	Prospective	2b	64	Nissen & Toupet	RA-ARS (32) vs L-ARS (32)	NR

^a^All studies were retrospectively staged into IDEAL stages and not provided by any authors, *RA* Robotically assisted, *L* Laparoscopic anti-reflux surgery, *ARS* Anti-reflux surgery, *NR* Not reported, *N/A* Not applicable to case report/series

*CR* Case report, *CS* Case series, *Co* Cohort study, *RCT* Randomised controlled trial

**Fig. 2:**
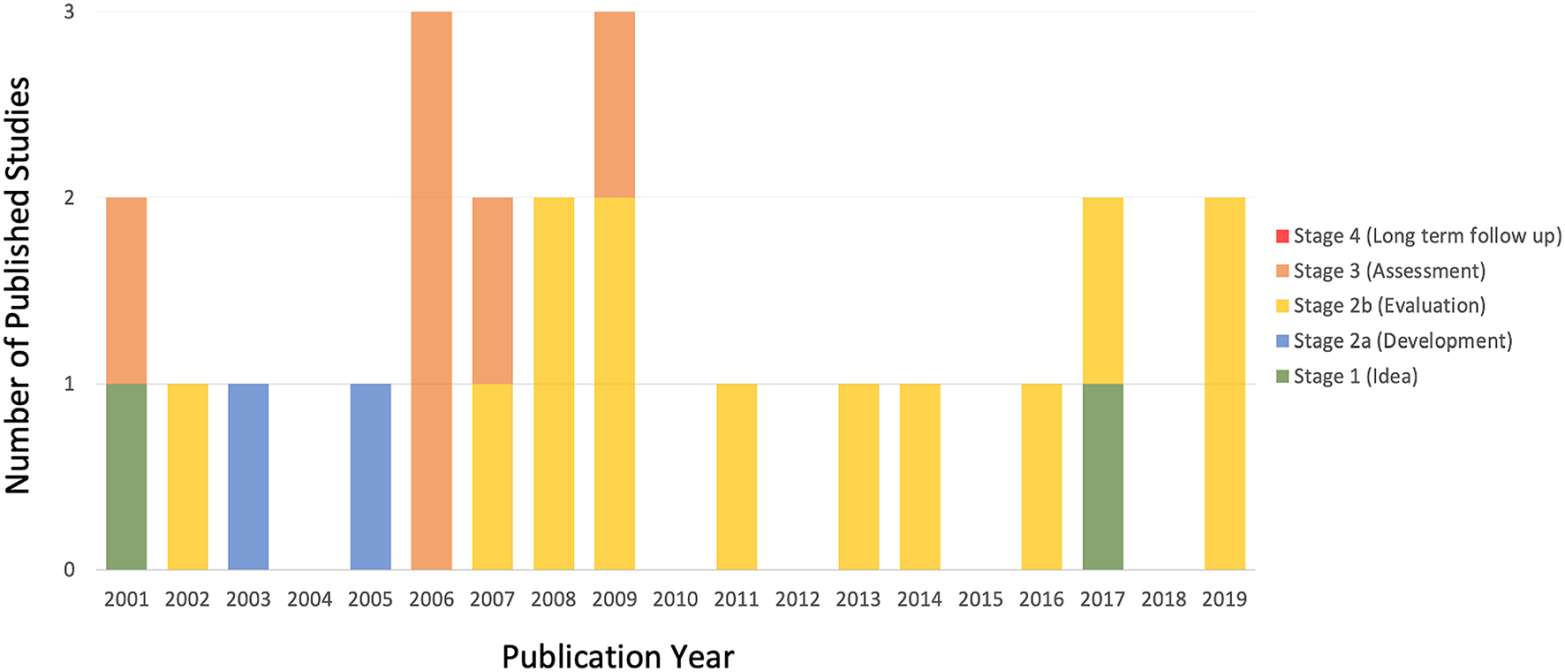
Bar chart showing the publication dates and IDEAL stages of included studies

No study reported an IDEAL stage. In addition to the first published case report [4], one further study was retrospectively classified as IDEAL stage 1 as it described the application of RA-ARS to a novel patient group (scleroderma oesophagus) [36]. Two studies were classified as IDEAL stage 2a (9%), 13 as stage 2b (57%) and six as stage 3 (26%). No studies were classified as stage 4. Sequential progression through the IDEAL stages over time was lacking: the first case report [4] and the first RCT [15] were published in the same year (2001), and all RCTs were published by 2009, with all subsequent studies either stage 2b (*n* = 7) or stage 1 (*n* = 1; Fig. 2).

All six included RCTs were single-centre comparisons of RA-ARS and laparoscopic techniques, involving between 20 and 50 participants. Of these, one was deemed to have an overall high risk of bias, and in three others, there was a lack of clarity around sequence generation and allocation concealment. There were some concerns regarding reporting bias in all six papers due to lack of previously published protocols, and a lack of clarity around sequence generation and allocation concealment in four [5, 15, 27, 32]. In the RCT with a high risk of bias, there were additional concerns regarding bias due to missing data and measurement of post-operative outcomes [5].

### Patient selection and demographics

Eleven studies (48%) reported both inclusion and exclusion criteria, five (22%) reported solely inclusion or exclusion criteria, and seven (30%) studies did not report any patient selection criteria (Table 2). Among the studies reporting this information, there were 17 distinct patient selection criteria, none of which were reported as specifically relating to the robotic nature of the procedure. One study excluded the first 10 patients undergoing RA-ARS [30], stating that this was done to minimise learning curve effects. No studies included a statement about what happened to ineligible patients. There was no discernible pattern in terms of widening of inclusion criteria over time or with advancing IDEAL stage.

**Table 2:** Summary of patient selection criteria

	Inclusion criteria	*n* = 23 (%)	Exclusion criteria	*n* = 23 (%)
Patient related	PPI-refractory GORD symptoms	5 (21)	Oesophageal rupture	1 (4)
	Unwillingness to take PPI	1 (4)	Predominant extra-intestinal symptoms	1 (4)
Comorbidity related	Low ASA score^a^	1 (4)	Morbid obesity^a^	3 (13)
			High ASA score^a^	2 (8)
			Psychiatric diagnoses	1 (4)
Investigation related	Pathological acid exposure on oesophageal 24-h pH monitoring	6 (25)	Giant or paraoesophageal hernia Oesophageal motility problem	4 (17) 3 (13)
	Prescence of hiatal hernia	2 (8)		
	Normal motility on manometry testing	1 (4)		
	Hill grade of gastro-oesophageal junction valves	1 (4)		
Operative factors			Previous major abdominal surgery^a^	6 (25)
			Concomitant cholecystectomy^a^	1 (4)
Other			First 10 patients undergoing robotic anti-reflux surgery	1 (4)

^a^Inclusion/exclusion criteria that could potentially be related to the robotic nature of the procedure (although not explicitly stated). *PPI* Proton pump inhibitor, *GORD* Gastro-oesophageal reflux disease, *ASA* American society of Anaesthesiology

A total of 28 demographic characteristics were identified across the included studies, including sex (*n* = 22, 96%), age (*n* = 21, 91%), body mass index (*n* = 12, 52%) and grade of oesophagitis (*n* = 7, 30%; Table 3). No demographic characteristics were reported across all studies, and 12 were reported only once. There was no evidence of widening of patient demographics (e.g. inclusion of older, more comorbid patients) over time or with advancing IDEAL stage.

**Table 3:** Patient demographic characteristics reported in the included studies

			*n* = 23 (%)
Intrinsic patient characteristics		Sex	22 (96)
		Age	21 (91)
		Race	2 (9)
Disease related characteristics		Number of years suffering with GORD	4 (17)
		Pre-op antisecretory medication use	2 (9)
		Diagnosis	1 (4)
		Gastrointestinal symptoms rating scale score	1 (4)
Comorbidity-related characteristics		Body mass index	12 (52)
		ASA grade	6 (26)
		Past surgical history	3 (13)
		Comorbidity (unspecified)	2 (9)
		Past medical history	1 (4)
		Risk of mortality	1 (4)
Investigation-related characteristics	Endoscopy	Grade of oesophagitis	7 (30)
		Prescence of hiatal hernia	2 (9)
		Hill grade	1 (4)
		Barrett’s metaplasia on endoscopy	1 (4)
		Endoscopy result	1 (4)
		Visick grade	1 (4)
	Oesophageal manometry	Lower oesophageal tone (mmHg)	6 (26)
		Distal oesophageal amplitude	2 (9)
		Oesophageal manometry	2 (9)
	pH studies	DeMeester score	6 (26)
		Acid monitoring studies	4 (17)
		Number of refluxes	1 (4)
		Number of refluxes lasting >5 min	1 (4)

*GORD* Gastro-oesophageal reflux disease, *ASA* American society of Anaesthesiology

### Governance and ethical factors

Fourteen articles (74%) reported institutional review board (IRB) or ethics committee approval. Three authors reported exemptions, although reasons were not provided. Fifteen studies (65%) reported obtaining consent from the included patients, of which one specifically documented the innovative nature of the RA-ARS. Sixteen articles (70%) did not include statements regarding COI. Two COI were declared: one author founded a robotics company [35], and another received honoraria for speaking on behalf of device companies[38]. Seventeen studies (74%) did not report whether funding was received and four (17%) stated that no funding was provided. One study received funding from the authors’ local department [36], and one from a medical device company [6].

### Surgeon expertise and training

Four studies (17%) described pre-specified criteria for surgeons to be eligible to participate in the study; in all cases, these criteria pertained to prior surgical experience. Thresholds of 10 [25], 20 [5], and 30 cases[27] were used for prior robotic experience and 30 cases [5, 32] for prior laparoscopic experience. No study reported specific training for surgeons prior to their first RA-ARS procedure.

Although 14 studies (61%) mentioned the number of operating surgeons (range, 1–3; median, 1), the majority (*n* = 20, 87%) did not report their grade or the previous number of RA-ARSs they had performed. In 13 studies, phrases such as ‘experienced’ or ‘senior’ were included as general statements of the operating surgeon’s experience.

Five studies (22%) measured and displayed the surgeons’ learning curve graphically. All five compared one or more surrogate markers for performance (operation time [*n* = 5], docking time [*n* = 2], complication rate [*n* = 1], length of stay [*n* = 1], console time [*n* = 1] and setup time [*n* = 1]), to the number of operations.

### Outcome selection, measurement and reporting

**Fig. 3:**
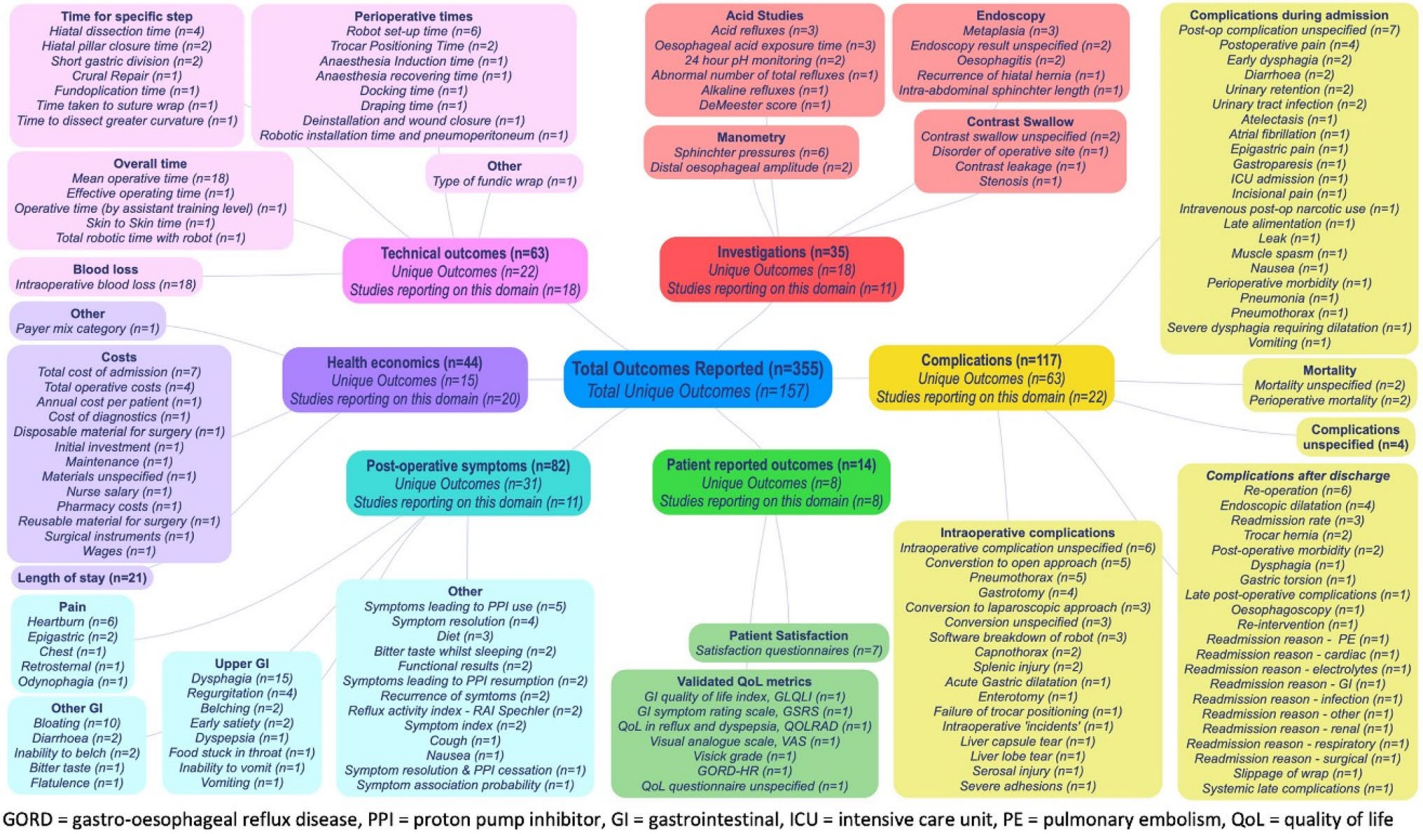
Diagram displaying all reported outcomes across all included studies

**Fig. 4:**
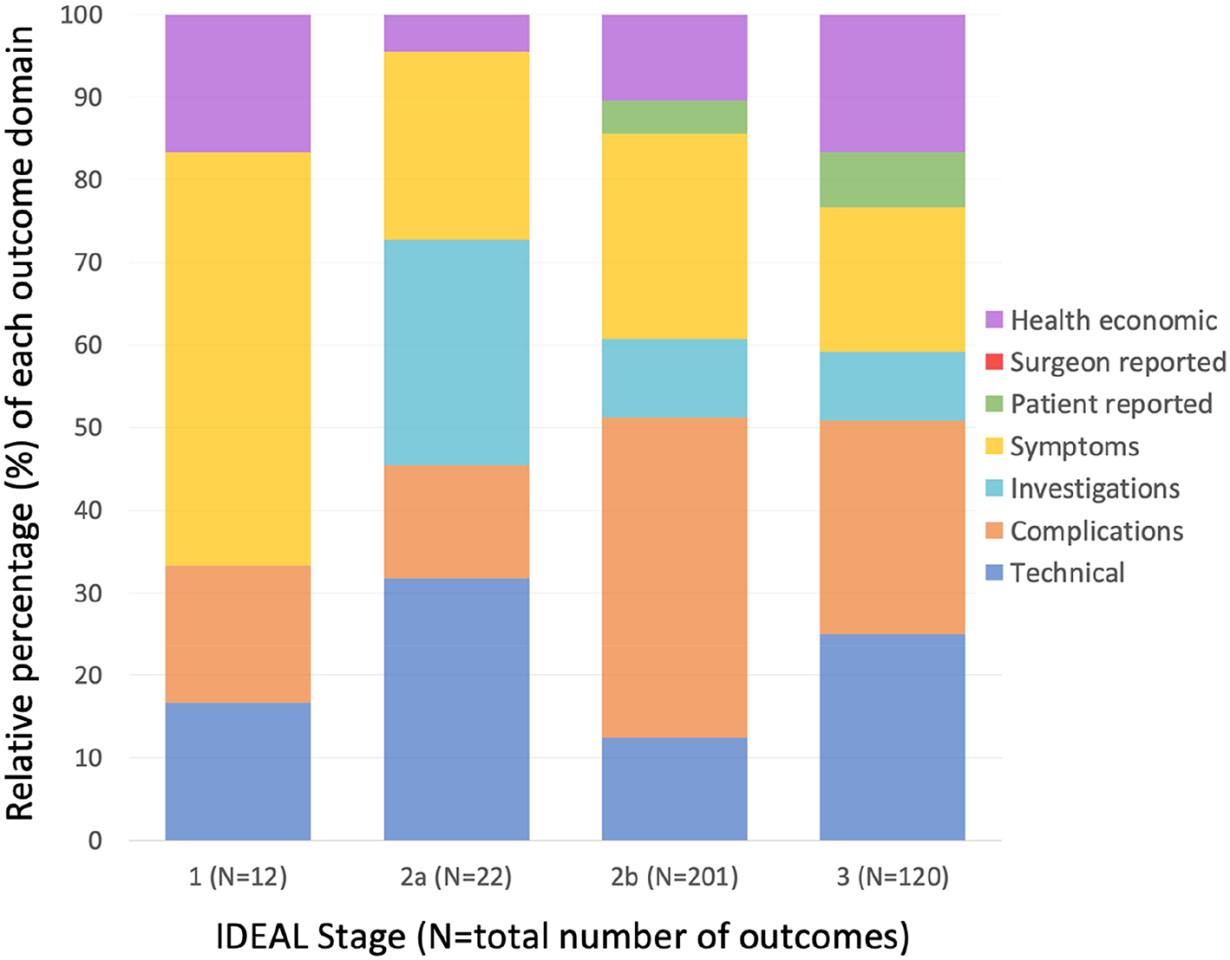
Bar chart showing papers’ reported outcomes by IDEAL stage

There were 157 distinct outcomes across the 23 studies, of which 95 (61%) were reported only once (summarised in Table 4, detailed in full in Fig. 3). No single outcome was reported in all 23 studies. The most frequently reported outcome domain was ‘complications’: 22 (96%) studies reported outcomes from this domain, and a third of all reported outcomes were from this domain (*n* = 117). The most frequently reported outcome was ‘mean operative time’ (*n* = 18, 78%), although there were 19 other different ways of reporting ‘time’, most of which were reported only once (*n* = 15, 79%). No study cited any surgeon-reported outcomes. The length of follow-up was reported in 15 (65%) studies (range 1–85 months; median, 24 months), of which 7 studies (47%) reported a follow-up period of less than one year. Contrary to the IDEAL recommendations, there was a lack of progression in the type of outcomes included (i.e. from technical to patient-reported outcomes) between each subsequent IDEAL stage (Fig. 4).

**Table 4:** Summary of outcome selection by domains, across included studies

Outcome domain	Total number of outcomes	Number of distinct outcomes	Number of studies reporting any outcomes in this domain
Technical	63	22	18
Complications	117	63	22
Investigations	35	18	11
Symptoms	82	31	20
Patient-reported	14	8	8
Surgeon-reported	0	0	0
Health economic	44	15	20
Total	355	157^a^	

^**a**^Of these, 97 were only reported by a single study

## Discussion

To our knowledge, this is the first study to undertake a detailed examination of the reporting of the introduction and evaluation of robotic anti-reflux surgery. The overall quality and consistency of reporting was deficient across the included studies. Outcome reporting was heterogeneous, with over half of all outcomes used only once across the studies. Patient selection criteria were variable, inconsistent, and sometimes omitted. Most studies did not provide statements about conflicts of interest and many did not report obtaining consent or ethics committee approval. The evolution of RA-ARS differed significantly from the IDEAL model of surgical innovation with a lack of stepwise progression from IDEAL stage 1 (Idea) to stage 4 (Long-term follow-up). The first RCT was published in the same year as the first case report, and the other five RCTs followed soon after, with most subsequent studies classified as stage 2b. Collectively, these findings suggest that there has not been sequential and incremental building of evidence from one study to the next.

Four previous meta-analyses have summarised the efficacy of RA-ARS, published in 2010 [41–43] and 2012 [44], which all included the six RCTs identified in this review. The methodological limitations of the included RCTs were highlighted in all reviews: small sample sizes from single centres, and a lack of information about randomisation, raising questions about the validity and reliability of the findings. Although one review suggested that postoperative complications might be reduced with RA-ARS, the authors agreed with findings from two other reviews that the advantages of robotic surgery did not translate to improved patient outcomes, with higher costs and longer operating times. Despite the same findings, the fourth review argued longer operating times were related to lack of familiarity with RA-ARS and concluded that RA-ARS was safe, effective and should be the ‘future trend for treatment of GORD’ [43]. Another problem was that the RCTs did not report the same outcomes, reducing the number of studies available for meta-analysis. Moreover, all six RCTs were undertaken early in the emergence of RA-ARS as a promising technique, raising the possibility that the results were influenced by learning curve effects. Were a well-designed, multicentre RCT to be conducted in the present day, it is possible that the findings would differ, and further research is therefore warranted in this area.

Although this was a comprehensive literature review, there were some limitations. Non-English language studies were excluded, meaning that valuable data may have been missed, although this has been shown not to cause systematic bias [18]. We excluded studies related to giant or paraoesophageal hernias because of differences in technique, disease, and complication profiles. As reflux symptoms can also occur in these conditions, our study may therefore not represent the entirety of RA-ARS. A final limitation was that determining the stage of innovation of a report was sometimes difficult using the algorithm provided by the IDEAL Collaboration [19]. For example, distinguishing stages 2a from 2b was particularly problematic (mainly due to a lack of information about the technique of RA-ARS and whether this was evolving), and they may represent either end of a continuum [9]. Moreover, the algorithm did not encompass the expected differences in outcomes or patient selection criteria, which are key considerations in moving between the IDEAL stages.

This study found that the evolution of RA-ARS differed significantly from the model of surgical innovation proposed by the IDEAL Collaboration. This may be a consequence of slow adoption of the IDEAL framework. Despite the publication of practical guidance in 2016 [9], a subsequent systematic review found that the IDEAL framework was not widely implemented outside the membership of the IDEAL Collaboration [11]. Several factors hindering its adoption have been described, including lack of understanding of the recommendations or how to apply them, and difficulty in determining the stage of innovation as was the case in our study. An updated framework and reporting guidance have been published to address the deficit in understanding [12], and the IDEAL collaboration is designing a study to investigation barriers to implementing the framework [personal communication, IDEAL Collaboration, 24th May 2022]. Furthermore, our institution is developing a method for determining stage of innovation. Collectively this may inform future studies, and prevent future innovations evolving in a similar manner to RA-ARS. Another possibility is that the IDEAL model may not be representative of real-world surgical innovation. The IDEAL model was partly derived from theories of diffusion of innovations in the social sciences rather than from any empirical study of innovation in practice [8], and therefore may not align well with real-world events. This could further explain the discrepancy between the IDEAL model and how RA-ARS has evolved. Real-world surgical innovation is being studied in depth at our institution using case study methods with multiple qualitative data sources [45].

The reporting of patient selection criteria and outcomes was highly heterogeneous. This caused difficulties in comparing studies and synthesising evidence in systematic reviews and meta-analyses [46], which could be remedied by using a core outcome set (COS). A COS is an agreed minimum set of outcomes that should be reported in all studies of a specific disease (such as GORD) [21], ensuring reporting consistency. The development of a COS for GORD in adults may therefore play an important role in improving the quality of future RA-ARS studies. While one has been developed for the paediatric population [47], a COS for GORD in adults has not yet been published. More generally, a COS for the standardised evaluation of surgical innovation has been developed [48], aiming to reduce outcome heterogeneity in the reporting of new surgical procedures or devices in the future.

In conclusion, the under-reporting of important aspects of study design and high degree of outcome heterogeneity impedes the ability to draw meaningful conclusions from the body of evidence. There is a need for further well-designed randomised trials, alongside agreement about outcome selection, measurement, and reporting for future RA-ARS studies. Furthermore, we support the development of a core outcomes set for adult GORD, and the use of frameworks such as those published by the IDEAL Collaboration.

## Supplementary Information

Below is the link to the electronic supplementary material.

Supplementary file1 (DOCX 19 KB)

## Data Availability

Full datasets are not publicly available, but are available from the corresponding author on reasonable request.
