# Supplementary material for: A systematic review of robot-assisted anti-reflux surgery to examine reporting standards
Source: J Robot Surg. 2022 Sep 8;17(2):313–24. doi: 10.1007/s11701-022-01453-2 (PMC10076351; doi:10.1007/s11701-022-01453-2)
Supplement: Supplementary file 1 — Supplementary file1 (DOCX 19 KB) [file 11701_2022_1453_MOESM1_ESM.docx]

# **SUPPLEMENTARY TABLES (ONLINE ONLY)**

### *Supplementary Table 1: Table of search terms*

=1     robotics/ (59149)

2     robot assisted surgery/ (10309)

3     robotic surgical procedure/ or robotic surgical procedures/ (10842)

4     computer assisted surgery/ or surgery, computer-assisted/ (26636)

5     computer assisted surgery system/ (123)

6     robotic surgical device/ or robotic console/ or robotic navigation system/ or robotic surgical system/ or robotic catheter system/ or robotic neurological surgical equipment/ or robotic orthopedic surgical system/ or robotic hip surgery system/ or robotic knee surgery system/ or robotic spine surgical system/ (2223)

7     robotic surgical instrument/ or robotic endoscope/ or robotic needle driver/ or robotic retractor/ or robotic scissors/ or robotic forceps/ or robotic dissecting forceps/ or robotic grasping forceps/ or robotic sealing device/ or robotic stapler/ or robotic vessel sealer/ (257)

8     robot assisted.hw. (13167)

9     (robot* or telerobot* or tele-robot* or telesurger* or tele-surger*).ti,ab,kf,kw. (114490)

10     (computer assist* adj5 (operation* or procedur* or surger* or surgeon? or surgical*)).ti,ab,kf,kw. (6202)

11     (da Vinci? or davinci?).mp. (11945)

12     (remote adj2 (operation* or procedur* or surger* or surgeon? or surgical*)).ti,ab,kf,kw. (1528)

13     ("state of the art" adj (surger* or surgical*)).ti,ab,kf,kw. (218)

14     (Arthrobot* or Aesop* or Hugo* or Mako* or MiroSurge* or Monarch* or Neuroarm* or Probot* or Revo-I or ROBODOC).mp. (7961)

15     (Senhance* or Sina or Sofie or SPIDER or SPORT or SurgiBot or Telelap or Unimation or Puma or Versius or ZEUS).mp. (170516)

16     or/1-15 (332575)

17     gastroesophageal reflux/ (81168)

18     fundoplication/ (15181)

19     *digestive system surgical procedures/mt (3507)

20     (GERD or ((gastric* or gastro* or esophag* or oesophag* or gastroesophag* or gastrooesophag*) adj2 reflux*)).mp. (111086)

21     (fundoplicat* or fundo-plicat*).mp. (19384)

22     exp gastroesophageal reflux/ (89697)

23     ((gastric* or gastro* or esophag* or oesophag* or gastroesophag* or gastrooesophag*) adj2 regurgitat*).mp. (935)

24     ((antireflux or anti-reflux) adj5 (laparoscop* or operation* or procedur* or surger* or surgeon? or surgical*)).ti,ab,kf,kw. (9153)

25     ((nissen or rossetti or toupet or lind or (watson not jones) or besley or dor) adj5 (laparoscop* or operation* or procedur* or surger* or surgeon? or surgical*)).tw. (5267)

26     or/17-25 (124411)

27     16 and 26 (1253)

28     remove duplicates from 27 (991)

29     (conference abstract or conference review).pt. (3821741)

30     28 not 29 (691)

### *Supplementary Table 2: Table of definitions of outcome domains used to categorise reported outcomes in included studies*

| Outcome domain | Definition |
| --- | --- |
| Technical | Outcomes related to the operation or robot itself. This includes outcomes such as operative time and blood loss, but does not include intra-operative complications. |
| Complications | Any deviation from the normal operative course^a^. This included intra-operative complications including conversions, post-operative complications including readmission and reoperation, as well as mortality during the study period. |
| Investigations | Outcomes related to objective results from laboratory, endoscopic or radiographic examinations. |
| Symptoms | Outcomes related to the post-operative resolution or persistence of GORD symptoms (e.g. pain, dysphagia, bloating). |
| Patient reported outcomes | Instruments such as satisfaction questionnaires and validated quality of life questionnaires, that must be completed independently by the patient^b^. |
| Surgeon reported | Outcomes related to the surgeons’ subjective experience of the robotic technology. |
| Health economic | Outcomes relating to the cost or resource use of any aspect of the admission. This includes outcomes such as operative costs, wages and length of stay. |

GORD = gastro-oesophageal reflux disease. ^a^Dindo D, Demartines N, Clavien PA. Classification of surgical complications: a new proposal with evaluation in a cohort of 6336 patients and results of a survey. *Ann Surg.* 2004;240(2):205-213.; ^b^Doward LC, McKenna SP.
